# Obesity and Metabolic Traits after High-Fat Diet in Iberian Pigs with Low Birth Weight of Placental Origin

**DOI:** 10.3390/biology11101533

**Published:** 2022-10-19

**Authors:** Ana Heras-Molina, Natalia Yeste, José Luis Pesantez-Pacheco, Susana Astiz, Marta Vazquez-Gomez, Arianna Bettiga, Francesco Trevisani, Consolacion Garcia-Contreras, Sergio Luis-Lima, Anna Bassols, Esteban Porrini, Antonio Gonzalez-Bulnes

**Affiliations:** 1 Instituto Nacional de Investigación (INIA), Consejo Superior de Investigaciones Científicas (CSIC), Ctra. De La Coruña Km. 7, 28040 Madrid, Spain; 2 Faculty of Veterinary Medicine, Universidad Complutense de Madrid (UCM), 28040 Madrid, Spain; 3 Departament de Bioquímica i Biologia Molecular, Facultat de Veterinària, Universitat Autònoma de Barcelona, 08193 Cerdanyola del Vallès, Spain; 4 School of Veterinary Medicine and Zootechnics, Faculty of Agricultural Sciences, University of Cuenca, Cuenca 010220, Ecuador; 5 Nutrition and Obesities—Systemic Approaches Research Unit, National Institute of Health and Medical Research (INSERM), Sorbonne Université, CEDEX 12, 75571 Paris, France; 6 Division of Experimental Oncology, Urological Research Institute, IRCCS San Raffaele, 20132 Milano, Italy; 7 Department of Nutrition and Sustainable Animal Production, Estacion Experimental del Zaidin, Consejo Superior de Investigaciones Científicas (CSIC), 18008 Granada, Spain; 8 Department of Nephrology and Hypertension, IIS—Fundacion Jimenez Diaz, 28040 Madrid, Spain; 9 Departamento de Medicina Interna, Hospital Universitario de Canarias, 38320 San Cristóbal de La Laguna, Spain; 10 Departamento de Producción y Sanidad Animal, Facultad de Veterinaria, CEU Universidad Cardenal Herrera, CEU Universities, 46115 Valencia, Spain

**Keywords:** animal models, intrauterine growth restriction, obesity, translational medicine

## Abstract

**Simple Summary:**

Intrauterine growth restriction is an increasingly concerning issue for human pregnancies because of its perinatal and longer-term consequences on offspring health. We have explored, in a swine model, differences in the adult phenotype of offspring with low birth weight induced by maternal malnutrition or placental insufficiency.

**Abstract:**

Intrauterine growth restriction (IUGR) and later obesity and metabolic disorders have classically been associated with maternal malnutrition, but most cases of IUGR are related to placental insufficiency. The current study, using a swine model for IUGR and obesity, aimed to determine the interaction of birth weight (categorized as low birth weight [LBW] or normal birth-weight [NBW]) and postnatal diet (categorized as maintenance diet [MD] or fattening diet [FD]) on body weight, adiposity and metabolic traits. FD induced higher body weight and adiposity (both *p* < 0.0001), with higher fructosamine levels (*p* < 0.005) and a trend toward higher HOMA-β index (*p* = 0.05). NBW pigs remained heavier than LBW pigs during the early juvenile period (*p* < 0.005), but there were no differences at later stages. There were no differences in metabolic traits during juvenile development, but there were differences in adulthood, when LBW pigs showed higher glucose and lower insulin levels than NBW pigs (both *p* < 0.05). These results suggest that (a) FD allows LBW offspring to achieve similar obesity in adulthood as NBW offspring, and (b) glucose metabolism is more compromised in obese LBW than obese NBW pigs. The comparison of our data with previous studies highlights significant differences between offspring with LBW induced by maternal malnutrition or placental insufficiency, which should be considered when studying the condition.

## 1. Introduction

Most recent theories support that the current human pandemics of obesity and associated metabolic disorders are strongly determined by interaction among genetic predisposition, malnutrition of the conceptus during pregnancy and postnatal exposure to obesogenic diets [1]. Malnutrition of the conceptus during pregnancy causes intrauterine growth restriction (IUGR) and subsequent low birth weight (LBW). The worldwide incidence of IUGR in humans ranges between 7% and 15% depending on sociodemographic factors, and it reflects mainly maternal malnutrition. IUGR accounts for 800,000 neonatal deaths worldwide [2,3]. Surviving IUGR offspring are predisposed to obesity and metabolic disorders when they are given obesogenic diets after birth [4,5]. The prevalence of these traits is even higher in resource-limited areas, reflecting thrifty genotype and leptin resistance syndrome [6,7].

To promote human health and well-being, it is necessary to increase our knowledge about the interplay among genetics, prenatal programming and postnatal lifestyle [8]. Current knowledge on this topic has mainly been generated from human cohort studies, which frequently prevents deeper analysis of the mechanisms behind such changes. Hence, interventional studies with appropriate animal models are mandatory. The main models in experimental studies on prenatal programming of obesity and associated disorders involve rats, mice, hamsters and guinea pigs [9,10,11]. However, these models can differ substantially from humans in terms of cell and tissue biology, metabolic and endocrine pathways and developmental patterns and physiology of organs and systems [12,13].

Large animal models, mainly pigs, are rapidly emerging as more adequate biomedical models due to their many similarities with humans in anatomy and physiopathology of digestive, renal, metabolic and cardiovascular systems [14,15,16]. The Iberian pig is a powerful model for studies of obesity and associated disorders: it is naturally leptin resistant, it has a substantial appetite and can fatten rapidly, and it is predisposed to metabolic syndrome, resembling patients with these disorders [17,18]. Our group has developed an Iberian pig model in which the intrauterine exposure of conceptuses to maternal undernutrition increases the incidence of IUGR and alters development and metabolism during the early postnatal and juvenile periods, which, in turn, alters the adult phenotype [19].

Classically, IUGR has been associated with maternal malnutrition, but most cases of IUGR are caused by placental insufficiency in the absence of maternal malnutrition [20,21]. Placental insufficiency is a growing problem and is linked to different factors, including delays in childbearing age, inadequate lifestyle, stress, sedentary habits, pollution, alcohol and tobacco consumption, obesity, diabetes and preeclampsia. Most of these factors are strongly related to a contemporary lifestyle, so we can expect the prevalence of this disorder to increase.

Whether the phenotype of offspring affected by IUGR depends on whether the IUGR is of maternal or placental origin remains unclear. Few studies have examined offspring affected by IUGR from mothers that were adequately fed during pregnancy. Hence, in the current study, we aimed to examine how the interaction between postnatal obesity and IUGR caused by placental insufficiency (in the presence of adequate maternal nutrition) would influence the appearance of metabolic disorders and metabolic syndrome in offspring.

Pigs are an outstanding model of placental insufficiency, which is well characterized in the species [22]. Pork production has been made more profitable by increasing the number of piglets per litter. This increase in the number of offspring was obtained by selecting lines with higher ovulation rate but at the cost of a higher rate of IUGR [23]. This reflects that the ovulation rate increased without a concomitant increase in uterine size or capacity. The same phenomenon has occurred in Iberian pigs, where breeding efforts have increased litter size from 6–6.5 piglets to 9–9.5 piglets [19,24]. Uterine capacity refers to the number of fully formed fetuses that the uterus can support until farrowing, and competition for limited space and nutrients within the uterus can affect fetal growth [25]. Reduced utero-placental blood flow and/or angiogenesis is associated with IUGR [26]. Currently, 15–25% of swine newborns are affected by IUGR. Placental insufficiency, growth inhibition and embryo losses have been studied in Iberian pigs [27,28,29].

IUGR in swine is associated with increased risk of pre-weaning morbidity and mortality, low efficiency of feed utilization, permanent stunting of growth and development, and poor quality of carcass and meat [22,30]. Despite extensive efforts to understand and decrease the occurrence of IUGR, it remains a major problem in pig production. At the same time, swine models of placental insufficiency and IUGR, including the Iberian breed, are extensively used as preclinical models [11,19,31,32]. 

## 2. Materials and Methods

### 2.1. Animals and Experimental Procedure

**Figure 1:**
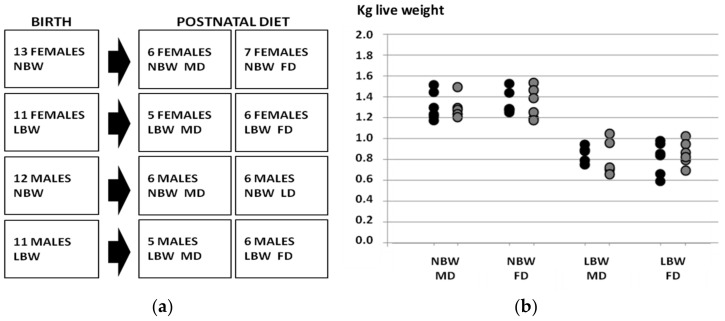
Study design (**a**) and birth weights of the piglets in the four groups of the study (**b**). NBW: normal birth weight; LBW: low birth weight; MD: maintenance diet; FD: fattening diet; black circles: males; grey circles: females.

The experiment involved 49 purebred Iberian piglets whose birth weight and sex distribution were representative of the breed (Figure 1). Thus, half of the piglets were selected within the normal range birth weight range for the Iberian breed (Group NBW, *n* = 25, 1.34 ± 0.02 kg), while the remaining piglets were selected as LBW (*n* = 24, 0.84 ± 0.02 kg; *p* < 0.0001 vs. NBW). Half of the piglets in the NBW and LBW groups were females and half were males, without significant differences in mean birth weight between males and females within each group. In each group, we did our best to assign one male and one female that were littermates from the same dam, but this was not always possible. As a result, piglets came from 16 instead of 12 sows. The 16 purebred Iberian sows, which had parities from 3 to 5, were synchronized with altrenogest (Regumate^®^, MSD Animal Health, Boxmeer, The Netherlands), then artificially inseminated with cooled semen from the same purebred boar. One female and one male from the LBW group died during the study, so ultimately 47 pigs were used in the study: 12 NBW females, 12 NBW males, 12 LBW females and 11 LBW males.

After weaning, at around 28 days of age, all piglets were housed in collective pens containing the same numbers of males and females. At the first month after weaning, the piglets were fed a standard diet with mean values of 18% of crude protein, 4.5% of fat and 3.35 Mcal/kg of metabolizable energy. Next, from 60 to 140 days of age, the piglets were fed a diet containing mean values of 15.1% of crude protein, 2.8% of fat and 3.08 Mcal/kg of metabolizable energy; the amount of food was adjusted for age in order to ensure daily maintenance requirements. Near infrared spectroscopy (NIRS) was used to determine the content of nutrients of feed, as routinely performed at the laboratories of the Faculty of Veterinary Medicine of the Universidad Complutense de Madrid.

From 140 to 385 days of age (adulthood), the pigs were allocated to different diets. Half of the females and males from each group continued on the same diet (maintenance diet, MD), containing 12.2% of crude protein, 2.8% of fat and 3.08 Mcal/kg of metabolizable energy. This amount of feed was increased to 2 kg/animal/day and served twice per day. The remaining pigs were allowed ad libitum access to the same diet but enriched in fat (6.3%), which therefore contained 3.36 Mcal/kg of metabolizable energy (fattening diet, FD). Food intake by the FD group was around 4 kg/animal/day from days 140 to 220, around 5 kg/animal/day from days 220 to 300 and around 6 kg/animal/day from days 300 until the end of the study. Composition and nutritional values are detailed in Supplementary Table S1.

Food was offered in individual feeders and consumption of the diet and refusals were checked individually for each pig and each meal. Refusals and loss of appetite were negligible during the study. 

The design of the experimental groups and birth weights in each group are depicted in Figure 1. The objective of the study was to assess the effects of sex, birth weight and postnatal diet on postnatal changes in body weight and size, adiposity, plasma indexes of glucose and lipid metabolism, antioxidant status and cardiovascular features.

### 2.2. Evaluation of Growth Patterns and Adiposity

In all pigs, changes in body weight and adiposity were recorded at 115, 140, 195, 250, 345 and 385 days of age. Body weights were used to calculate average daily weight gain (ADWG) in the intermediate periods of age and across lifetime. Adiposity was assessed in terms of subcutaneous back fat depth and intramuscular fat content. Subcutaneous back fat depth was determined at the P2 point, which lies on the right side of the animal at 4 cm from the midline and transversal to the head of the last rib. The fat depth at this point was measured using a SonoSite S-Series ultrasound machine with a linear array probe (5–8 MHz; SonoSite, Bothell, WA, USA). Intramuscular fat content was determined in samples obtained immediately after euthanasia, and fat was extracted after lyophilization and homogenization, as described elsewhere [33]. Fat content was calculated and expressed as a percentage.

### 2.3. Evaluation of Metabolic Indexes

Glucose parameters (glucose, fructosamine and insulin), lipid profiles (total cholesterol, high-density lipoprotein cholesterol [HDL-c], low-density lipoprotein cholesterol [LDL-c], and triglycerides) as well as non-esterified fatty acids (NEFA), total proteins and urea were measured using a clinical chemistry analyzer (Saturno 300 plus, Crony Instruments, Rome, Italy), according to the manufacturer’s instructions. Insulin was measured with a Porcine Insulin ELISA kit (Mercodia, Uppsala, Sweden); the manufacturer-specified assay sensitivity was 0.26 IU/L and intra-assay variation coefficient was 3.5%. Possible changes in β-cell function and insulin resistance (IR) during the experimental protocol were assessed through homeostasis model assessment (HOMA), using the equations HOMA-IR = (FINS × FPG)/22.5 to assess insulin resistance and HOMA-β = (20 × FINS)/(FPG − 3.5) to assess beta cell function. Fasting plasma insulin concentration (FINS) in U/L and fasting plasma glucose concentration (FPG) in mmol/L were also determined.

On the last day of the experimental procedure, when pigs were 385 days old, samples were assayed for cortisol and redox status based on levels of malondialdehyde (MDA, TBARS/TCA assay kit, Cayman Chemical, Ann Arbor, MI, USA), glutathione peroxidase (GPx) and superoxide dismutase (SOD). Both parameters were assayed using diagnostic kits manufactured by RANDOX (Randox Laboratories, Crumlin, Ulster, UK) with an AU400 automatic analyzer (Beckman Coulter, Hamburg, Germany).

### 2.4. Measurement of Cardiovascular Features

Blood pressure was monitored using a tail-cuff sphygmomanometer on the last day of sampling, when animals were 385 days old. Pressure was measured before blood samples were drawn.

### 2.5. Statistical Analysis

Changes in body weight, adiposity and metabolic features over time were measured using Pearson correlation. The effects of sex (female vs. male), birth weight (NBW vs. LBW) and postnatal diet (MD vs. FD) on these variables were assessed using split-plot, repeated-measures analysis of variance (ANOVA; SPSS 22.0 (IBM, New York, NY, USA)), after testing for normality of data using the Shapiro–Wilk test. Effects on adult phenotype, including cardiovascular features, were assessed using two-way ANOVA if variance was homogeneous based on Levene’s test; otherwise, the effects were assessed using the Kruskal–Wallis test. Differences among groups were further analyzed using a Duncan post hoc test. Results were expressed as mean ± SEM, and statistical significance was accepted if *p* < 0.05.

## 3. Results

### 3.1. Effects on Growth Patterns and Adiposity

**Figure 2:**
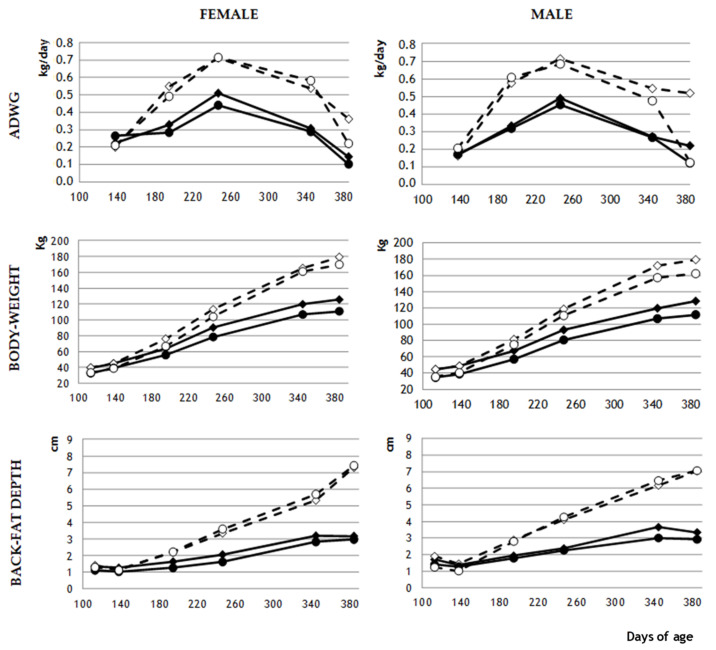
Mean values of average daily weight gain (ADWG; kg/day), body weight (kg) and back-fat depth (cm) in female and male pigs with normal birth weight (NBW; diamond marks) or low birth weight (LBW; circle marks) that were fed a maintenance diet (MD; continuous lines and filled marks) or a fattening diet (FD; discontinuous lines and empty marks). For clarity, SEM is not shown, but can be seen in Supplementary Table S2.

At 115 and 140 days of age, when all animals were fed an MD, NBW pigs were heavier than LBW pigs *(p* < 0.0001), and this did not depend on sex (Figure 2). Similarly, subcutaneous fat was deeper in NBW pigs than in LBW pigs (*p* < 0.005) at these two time points, independently of sex.

From day 195 until the end of the experiment, NBW and LBW males fed an MD did not differ significantly in body weight or adiposity. Among females fed an MD, NBW animals were significantly heavier than LBW animals on day 195 (*p* < 0.05), but subsequently NBW and LBW females did not differ significantly in body weight. Among MD animals, NBW females showed significantly higher adiposity than LBW females on days 195 (*p* < 0.005) and 250 (*p* < 0.05), but not later.

**Figure 3:**
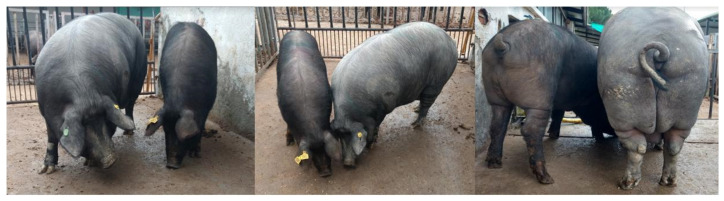
Corpulence and adiposity in pigs fed with the maintenance diet (MD) or fattening diet (FD).

The FD produced significant phenotypic differences from MD-fed animals from day 140 onwards (Figure 3). FD animals showed significantly higher ADWG (*p* < 0.0001), and therefore, higher body weight and subcutaneous adiposity (both *p* < 0.0001) throughout the experimental period (Figure 3). At the end of the study, intramuscular adiposity was also higher in FD animals (26.1 ± 2.6% vs. 12.8 ± 0.6%; *p* < 0.0001). Among male animals fed an FD, body weight and adiposity never differed significantly between NBW and LBW animals. Among female animals fed an FD, body weight and adiposity differed significantly between NBW and LBW animals only on day 195 (*p* < 0.05), while neither subcutaneous nor intramuscular adiposity differed significantly throughout the study.

### 3.2. Effects on Metabolism and Redox Status

At 115 and 140 days of age, when all animals were fed an MD, there were no significant differences in any metabolic parameters analyzed (Supplementary Table S3). From day 140, when some animals were given an FD, metabolic differences began to emerge; these differences were not significantly affected by birth weight or sex.

On day 195, FD pigs showed higher values of fructosamine (*p* < 0.005), triglycerides (*p* < 0.05), total cholesterol (*p* < 0.0005), HDL-c (*p* < 0.005), LDL-c (*p* < 0.0001), and urea (*p* < 0.0001) than MD pigs. At the next assessment, on day 250, the difference in fructosamine was no longer significant, but the differences remained significant for triglycerides (*p* < 0.0005), total cholesterol (*p* < 0.0005), HDL-c (*p* < 0.05), LDL-c (*p* < 0.0005), urea (*p* < 0.0001) and NEFA (*p* < 0.005). On day 345, differences were still significant for triglycerides (*P* < 0.005) and total cholesterol (*p* < 0.005). At this time point, glucose was also significantly higher in FD animals.

On day 385, when animals were adults, the FD group maintained higher levels of total cholesterol (*p* < 0.001), triglycerides (*p* < 0.01) and cortisol (*p* < 0.05) than the MD group (Table 1). The interaction between diet and birth weight exerted a significant influence on total protein (both *p* < 0.05) and showed a tendency to influence urea (*p* = 0.09).

Assessments of glucose parameters and indexes for insulin resistance (Table 2) indicated that FD pigs showed higher levels of fructosamine (*p* < 0.005) and a trend toward higher insulin levels (*p* = 0.09) and HOMA-β index (*p* = 0.05). Birth weight affected glucose metabolism independently of diet, with LBW pigs showing higher glucose and lower insulin levels than NBW pigs (both *p* < 0.05). On the other hand, birth weight did not affect HOMA-IR or HOMA-β indexes.

Assessment of redox biomarkers on day 385 showed that the FD group had lower levels of SOD (*p* < 0.05), and that the interaction between diet and birth weight significantly influenced levels of GPx and MDA (both *p* < 0.05; Table 3).

### 3.3. Effects on Cardiovascular Features

Assessment of blood pressure at day 385 showed no significant differences due to birth weight or sex, but diet did have a significant influence: FD was associated with higher systolic pressure (155.3 ± 1.7 vs. 141.8 ± 1.9 mmHg *p* < 0.05), diastolic pressure (111.6 ± 1.8 vs. 103.4 ± 1.9 mmHg, *p* = 0.07) and mean pressure (125.9 ± 1.8 vs. 116.6 ± 1.8 mmHg; *p* < 0.05). 

## 4. Discussion

The results of the present study indicate different lifelong developmental trajectories depending on whether birth weight is normal or low as a result of placental dysfunction. The main findings of our study are that (a) an obesogenic diet in LBW animals allowed their body weight, corpulence and adiposity to “catch up” to those of NBW animals by the end of the experiment; and (b) despite this similar weight gain, obese LBW animals showed lower insulin levels and higher levels of glycemia than obese NBW animals.

### 4.1. Catch-Up Growth in Obese LBW Animals

The differences between LBW and NBW animals were independent of sex during early development, although significant differences emerged between the sexes in adulthood. Body weight and adiposity were lower in female and male LBW offspring than in NBW offspring from weaning until day 195 of age. These differences disappeared in early adulthood, first among males and later among females, regardless of whether animals were fed an MD or FD. In males and females, an FD during the juvenile period induced higher body weight and adiposity than an MD. An FD also allowed LBW animals to achieve body weight and adiposity similar to NBW animals at an earlier age (day 180 for males, day 195 for females) than MD allowed (day 195 for males, day 250 for females). These features indicate catch-up growth in LBW offspring, especially in males.

Previous studies on Iberian pigs have linked maternal undernutrition to higher incidence of LBW in offspring [34,35]. Our LBW neonates, whose LBW resulted from placental insufficiency, had the same phenotype as LBW neonates in those two previous studies, whose LBW resulted from maternal malnutrition. However, feeding an FD to LBW piglets in the present study led to a quite different developmental trajectory than feeding an FD to LBW piglets in the previous studies. The LBW piglets in the present study took longer than those in the previous ones to increase in body weight and adiposity. In addition, the effects of an FD were stronger in females than males in those previous studies, reflecting the stronger predisposition to obesity among females whose LBW is due to maternal undernutrition. In fact, in those previous studies, although LBW females reached similar weight and size as NBW females during lactation, LBW males continued to be smaller than NBW males.

These findings suggest that maternal undernutrition and placental insufficiency affect prenatal programming differently. Although both events are related to IUGR, catch-up growth in LBW offspring occurs significantly earlier in development when the LBW is due to maternal undernutrition. This implies that maternal undernutrition stimulates more strongly compensatory mechanisms to compensate IUGR [36]. The possible existence of signaling based on maternal nutritional state is supported by the finding that males have an enhanced catch-up growth in case of placental IUGR whilst such enhanced postnatal development is more evident in females in cases of maternal IUGR. These findings may be consistent with the fact that, in humans and mouse models, females show better survival than males in the face of malnutrition [37,38,39,40,41].

The survival advantage of females in the face of maternal malnutrition appears to be even stronger in the presence of a thrifty genotype [42]. The survival advantage may reflect higher availability of glucose, cholesterol and essential fatty acids [43,44]. Our observation of a male advantage in catch-up growth under conditions of placental insufficiency rather than maternal undernutrition supports the importance of maternal nutrition in prenatal programming. It may be that developmental programming in female piglets evolved to be more resistant to maternal malnutrition than programming in males, given the importance of females for species survival. Future research should explore this possibility.

### 4.2. Metabolic Syndrome in Obese LBW Animals

Catch-up growth is beneficial to offspring to a certain point, but in an obesogenic environment, it may be harmful because it can lead to obesity, hyperleptinemia, hyperinsulinism and hypertension in adulthood [45,46,47,48]. Indeed, pigs on an FD in our study showed significant differences in glucose, triglycerides and cholesterol from pigs on an MD from day 195 of age. These differences increased with age and, at the last sampling at day 385, pigs on an FD showed higher levels of cholesterol, triglycerides and fructosamine, and they tended to show higher insulin levels and HOMA-β index. These changes reflect impaired glucose regulation: insulin secretion had to increase in order to maintain adequate plasma glucose concentrations, despite high fructosamine concentrations, as observed in humans [49]. This altered glucose regulation may reflect increases in fat content, since increased adiposity in humans increases insulin resistance [49,50]. Despite the increased insulin resistance, no pig in our study developed overt diabetes, perhaps because we did not observe the animals until older age or because pancreatic β-cells have a greater capacity to increase insulin secretion [16,51,52].

In addition, our pigs on an FD showed higher plasma levels of cholesterol and triglycerides, both of which indicate metabolic syndrome usually found at more advanced ages [53]. Hypertriglyceridemia is primarily related to the amount of visceral fat and may indicate glucose resistance [54,55,56]. In fact, the simultaneous increases in insulin resistance and plasma triglycerides in the current study support a link between insulin resistance and elevated triglycerides in blood and tissues [57,58,59,60].

Hence, pigs on an FD in our study clearly exhibited three of the five symptoms of metabolic syndrome: obesity, impaired glucose regulation, and insulin resistance. Since three of these five symptoms are sufficient to diagnose humans with metabolic syndrome [61,62,63,64], we conclude that our swine model reasonably recapitulates metabolic syndrome. Independently of diet, birth weight significantly affected parameters related to glucose metabolism: LBW animals showed higher glucose and lower insulin than their NBW counterparts. However, obese LBW animals did not show higher insulin levels than their non-obese counterparts, in contrast to the increase observed in obese NBW animals. This contrast suggests an association between LBW and islet damage, which prevents the increase in insulinemia that normally occurs in obesity. Indeed, this association makes LBW in humans a risk factor for type 2 diabetes. In our swine model, feeding LBW offspring an FD exacerbated the metabolic and redox effects of LBW, leading to higher levels of cholesterol, total protein, and biomarkers of oxidative stress (GPx and MDA).

## 5. Conclusions

We present a large animal model of obesity and metabolic syndrome related to birth weight and diet. This model may prove useful for fundamental and translational studies about the effects of such a phenotype on health during development and in adulthood. Our study uncovers significant differences in developmental trajectories between animals whose LBW is due to maternal malnutrition or placental insufficiency, which should be considered when studying the condition. Our study also uncovers substantial sex differences in the effects of LBW and eating an FD, highlighting the importance of maternal nutrition in prenatal programming. The possibility that female offspring are better equipped to resist nutritional scarcity during development should be explored further.

## Figures and Tables

**Table 1:** Mean (± S.E.M.) and *p* values for plasma concentrations of metabolic parameters on day 385 of age in female and male pigs born with normal birth weight (NBW) or low birth weight (LBW) that were fed a maintenance diet (MD) or fattening diet (FD).

Parameter		NBW	LBW	*p*-Value		
				Birth Weight	Diet	Interaction
Cholesterol (mg/dL)	MD	104.6 ± 7.00	102.64 ± 7.02	0.326	0.0008	0.124
	FD	126.98 ± 3.24	118.09 ± 6.64			
HDL-C (mg/dL)	MD	33.62 ± 3.45	33.08 ± 5.67	0.864	0.226	0.660
	FD	35.57 ± 4.04	35.97 ± 6.07			
LDL-C (mg/dL)	MD	63.21 ± 5.10	64.05 ± 10.06	0.647	0.129	0.347
	FD	70.53 ± 8.24	68.42 ± 9.97			
Triglycerides (mg/dL)	MD	54.91 ± 3.47	55.80 ± 5.21	0.126	0.005	0.276
	FD	96.5 ± 8.82	71.12 ± 7.76			
NEFAS (mM)	MD	0.30 ± 0.03	0.32 ± 0.03	0.412	0.861	0.801
	FD	0.30 ± 0.04	0.33 ± 0.03			
Total protein (g/dL)	MD	6.99 ± 0.19	7.66 ± 0.27	0.239	0.240	0.022
	FD	7.21 ± 0.12	6.99 ± 0.19			
Urea (mg/dL)	MD	18.29 ± 1.59	22.29 ± 2.79	0.628	0.770	0.093
	FD	21.94 ± 1.30	19.71 ± 1.58			
Cortisol (ng/mL)	MD	64.69 ± 12.45	70.52 ± 9.07	0.190	0.039	0.391
	FD	80.53 ± 11.51	107.99 ± 15.93			

HDL-c, high-density lipoprotein cholesterol; LDL-c, low-density lipoprotein cholesterol; NEFA, non-esterified fatty acids.

**Table 2:** Mean (± S.E.M.) and *p*-values of plasma concentrations for parameters of glucose metabolism, at 385 days-old, in female and male pigs with normal or low birth-weight (NBW and LBW, respectively) and fed with maintenance or fattening diets (MD and FD, respectively).

Parameter		NBW	LBW	*p*-Value		
				Birth Weight	Diet	Interaction
Glucose (mg/dL)	MD	89.27 ± 5.36	96.04 ± 3.41	0.033	0.837	0.412
	FD	84.20 ± 2.37	99.08 ± 6.76			
Fructosamine (mg/dL)	MD	314.09 ± 6.65	321.90 ± 8.82	0.447	0.004	0.284
	FD	347.92 ± 5.60	347.00 ± 7.72			
Insulin (mU/L)	MD	19.56 ± 2.23	16.55 ± 1.95	0.009	0.091	0.118
	FD	28.16 ± 3.56	16.91 ± 1.83			
HOMA-IR	MD	4.33 ± 0.56	4.16 ± 0.53	0.686	0.657	0.640
	FD	4.60 ± 0.41	4.35 ± 0.39			
HOMA-β	MD	228.35 ± 37.74	195.62 ± 29.57	0.899	0.053	0.211
	FD	321.31 ± 21.95	333.77 ± 67.09			

HOMA-IR, homeostasis model for estimating insulin resistance; HOMA-β, homeostasis model for estimating β-cell function.

**Table 3:** Mean (± S.E.M.) and *p*-values of plasma concentrations of parameters for oxidative status, at 385 days-old, in female and male pigs with normal or low birth-weight (NBW and LBW, respectively) and fed with maintenance or fattening diets (MD and FD, respectively).

Parameter		NBW	LBW	*p*-Value		
				Birth Weight	Diet	Interaction
MDA (µM)	MD	1.78 ± 0.09	1.41 ± 0.09	0.612	0.757	0.016
	FD	1.51 ± 0.11	1.75 ± 0.16			
GPx (U/L)	MD	962.53 ± 41.77	1114.29 ± 78.03	0.464	0.401	0.030
	FD	1119.78 ± 38.77	1042.91 ± 45.13			
SOD (U/mL)	MD	0.26 ± 0.02	0.22 ± 0.04	0.660	0.029	0.410
	FD	0.16 ± 0.03	0.17 ± 0.03			

MDA: malondialdehyde; GPx: glutathione peroxidase; SOD: superoxide dismutase.

## Data Availability

All data are included in the manuscript and Supplementary Tables.

## Supplementary Materials

The following supporting information can be downloaded at: https://www.mdpi.com/article/10.3390/biology11101533/s1, Table S1. Ingredient composition and nutritional analysis (g/kg, dry-matter basis) of experimental diets of pigs; Table S2. Mean values (± S.E.M.) of average daily weight gain (ADWG; kg/day), body weight (kg) and back-fat depth (cm) in female (F) and male (M) pigs with normal birth-weight (NBW) or low birth weight (LBW) and fed with maintenance diet (MD) or fattening diet (FD); Table S3. Mean values (± S.E.M.) for biochemical parameters of pigs with normal birth-weight (NBW) or low birth weight (LBW) and fed with maintenance diet (MD) or fattening diet (FD).
